# Auricular cartilage flap for device salvage in revision cochlear implant surgery

**DOI:** 10.1007/s00405-025-09491-w

**Published:** 2025-05-30

**Authors:** Emirhan Ceviken, Serdal Celik, Merve Torun Topcu, Mahmut Tayyar Kalcioglu

**Affiliations:** 1 Department of Otorhinolaryngology, Faculty of Medicine, Istanbul Medeniyet University, Istanbul, Türkiye; 2 ENT Clinic, Goztepe Suleyman Yalcin City Hospital, Istanbul, Türkiye; 3 Department of Audiology, Faculty of Health Sciences, Istanbul Medeniyet University, Istanbul, Türkiye

**Keywords:** Cochlear implant, Revision, Cartilage-reinforced flap

## Abstract

Although the number of cochlear implant surgeries performed to restore hearing in people with hearing loss is increasing with the expansion of indications, the likelihood of complications and revision surgeries is decreasing proportionally as surgical techniques and device technology have evolved over the years. In this case, a cartilage-reinforced flap approach was used to solve the problem of a patient whose implant electrode became visible under the skin five years after the initial surgery. Since it was not possible to replace the implant during revision surgery due to financial reasons, this surgical approach was preferred to preserve the device. In certain situations it may be necessary to develop solutions outside of established protocols. In this case, while the standard approach would be to remove the implant, create a new implant bed and perform a revision surgery with a new implant, the patient’s inability to obtain a new device necessitated the preservation of the existing implant. This approach successfully resolved the problem and prevented the patient from losing access to sound.

## Introduction

Cochlear implants are the most effective devices for restoring hearing in severe and profound sensorineural hearing loss [1]. Over the years, the indications have expanded and the number of cases performed has increased. With the widespread use of cochlear implants, the number of reported revisions has also increased [2]. Despite the high cost, most complications require reimplantation. This case report describes the salvage of the electrode fistulized to the skin from the posterior auricle and triangular fossa using an auricular cartilage rotation flap in a patient without financial support to receive a new device for reimplantation.

## Case presentation

The patient, born with severe bilateral sensorineural hearing loss, underwent bilateral cochlear implantation using the Nucleus Cl422 model in our clinic at the age of four years after failing to achieve adequate benefit from hearing aids. Intraoperative telemetry measurements were within normal limits, and Neural Response Telemetry (NRT) responses were recorded for all electrodes bilaterally. Postoperative telemetry measurements showed that bilateral electrode impedances remained within normal limits. NRT thresholds for the 1st, 11th, and 22nd electrodes were 187-145-178 for the right ear and 181-147-151 for the left ear, respectively.

Postoperative, bilateral free-field audiometric thresholds at 250, 500, 1000, 2000, 4000, and 6000 Hz were 35-40-40-35-40-45 dB HL, respectivrely, and the speech detection threshold was 35 dB HL. The speech recognition test could not be performed due to insufficient speech development.

The patient who continued auditory-verbal therapy underwent postoperative follow-up. With auditory-verbal development, the final evaluation revealed a bilateral speech detection threshold of 35 dB HL and a speech discrimination score of 64%.

When the performance of the cochlear implant was assessed separately for the right and left ears, the free-field pure-tone audiometric thresholds were as follows. Right ear: 35-35-30-35-35-40 dB HL at 250, 500, 1000, 2000, 4000, and 6000 Hz, respectively. Left ear: 30-35-30-35-35-35 dB HL at 250, 500, 1000, 2000, 4000, and 6000 Hz, respectively.

Speech detection thresholds were measured at 35 dB HL for the right ear and 30 dB HL for the left ear. Speech discrimination scores were recorded as 60% for the right ear and 64% for the left ear.

**Fig. 1:**
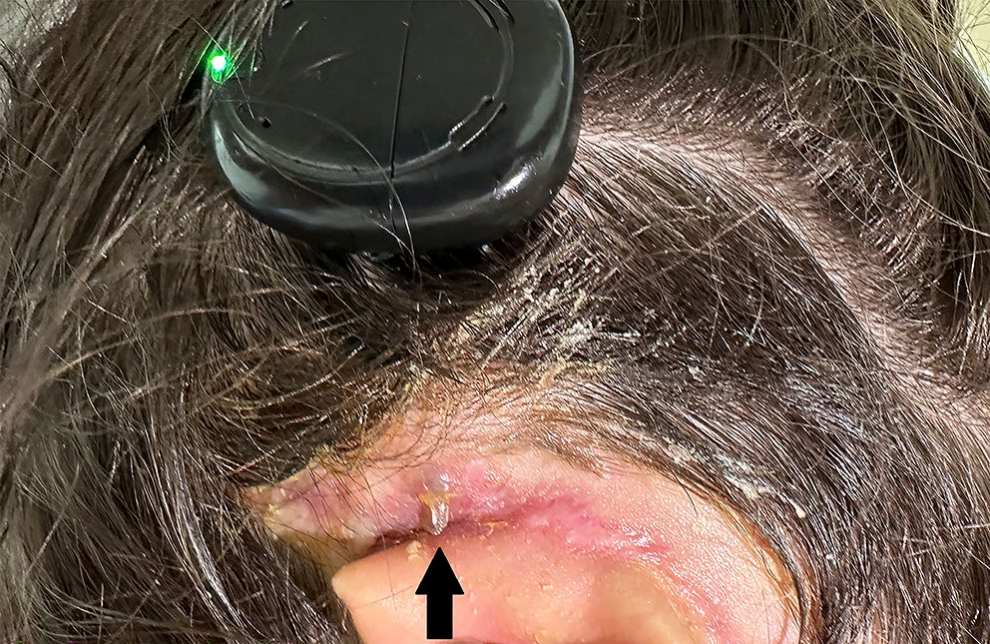
The ground electrode visible under the skin

With no problems noted during follow-up and development of speech and hearing, the patient presented approximately five years postoperatively with complaints of the implant electrode becoming visible behind the left ear. Examination revealed that the ground electrode, located just above the main electrode, had become visible under the very thin skin (Fig. 1). The electrode was not completely exposed to the skin. This was the chance of this patient.

There were two options for this case. One was to remove the implant and use a new one. This was not possible for this patient as there were no financial resources to cover the cost of a new implant. In this case, the patient would have been deprived of hearing. The second option was to do something with the same implant to ensure that the patient would not be left without hearing, even if there was a risk of infection. We informed the child’s family and decided to use the same and still functioning implant for a while.

**Fig. 2:**
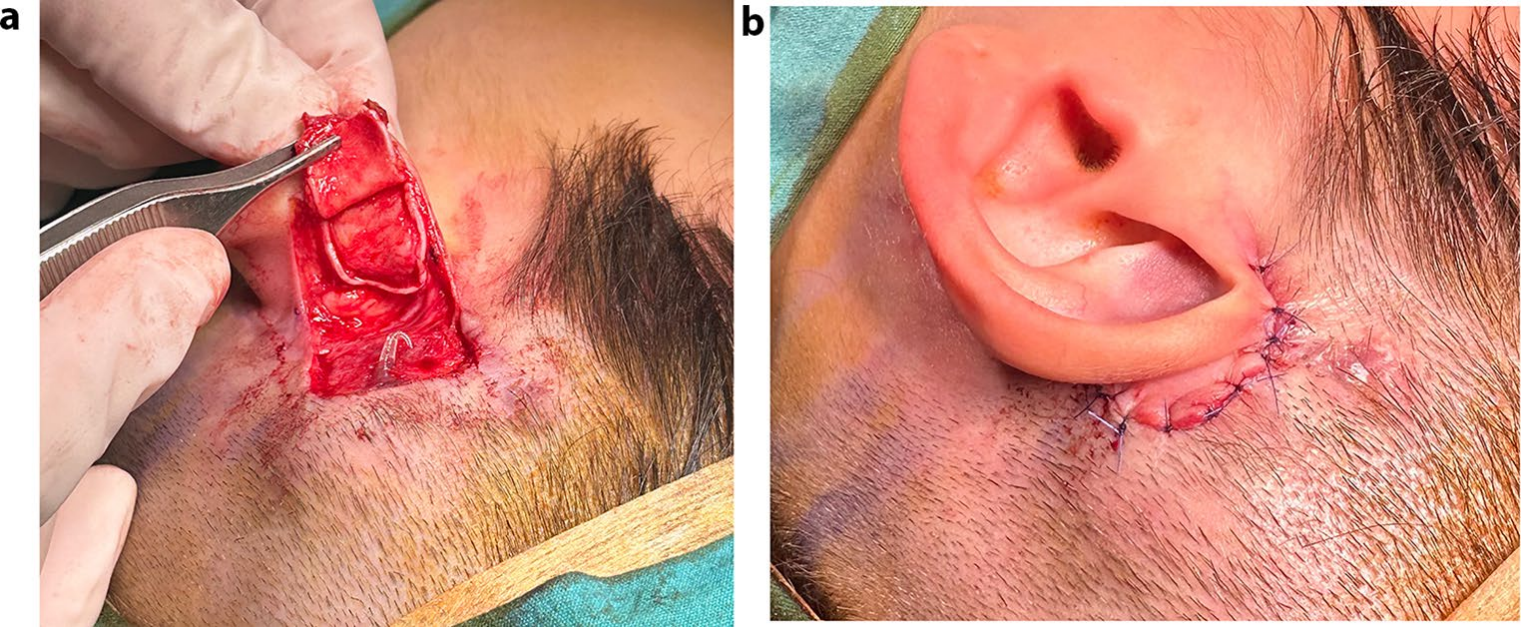
**a**: Single lobe cartilage flap prepared from the posterior auricle with skin. **b**: Closing the defect with the created flap

Due to the proximity of the defect, a cartilage-reinforced posterior auricular flap with skin graft was performed. Under general anaesthesia, a 1.5 × 1.5 cm excision of skin and subcutaneous tissue was performed around the ground electrode protruding through the skin. To close the defect, a cartilage-containing single-lobe flap was prepared from the posterior auricle and rotated to the defect (Fig. 2a). It was then primary sutured (Fig. 2b). The patient received antibiotics for 3 weeks. Early postoperative follow-up showed no complications.

**Fig. 3:**
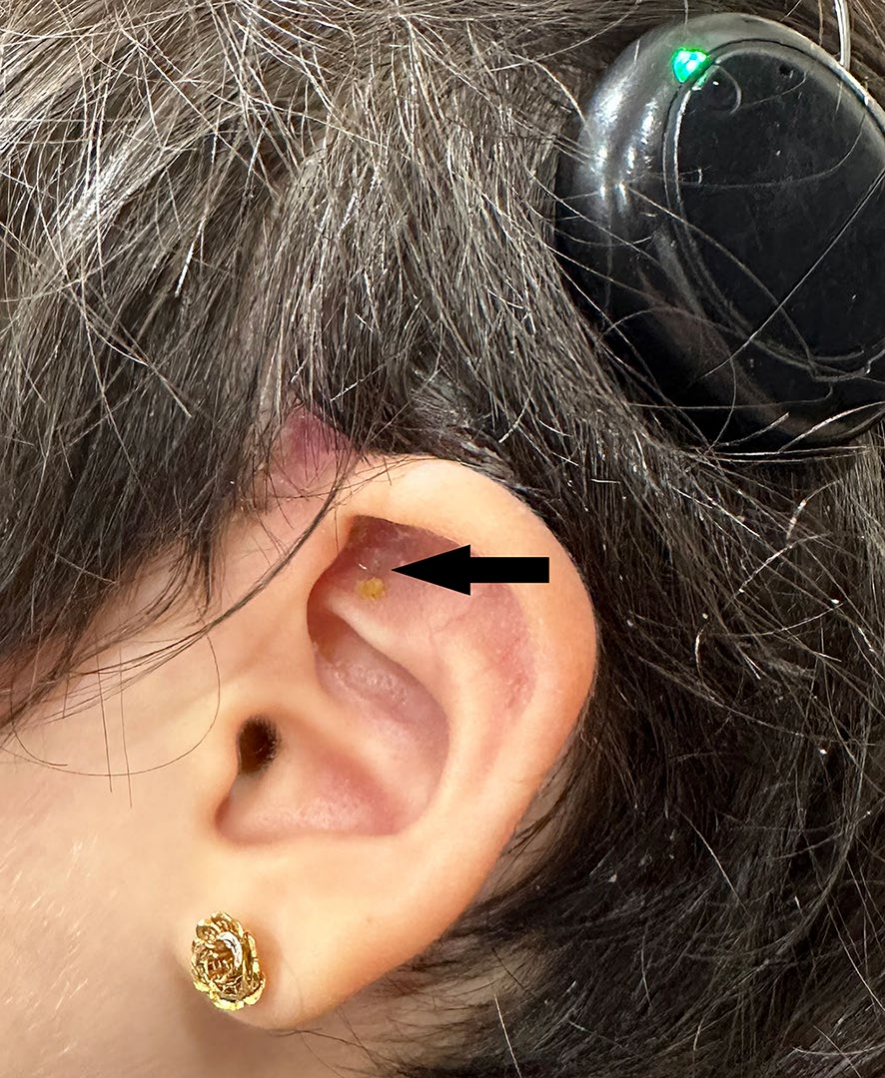
Electrode visible under the thinned tissue where the cartilage graft was harvested

**Fig. 4:**
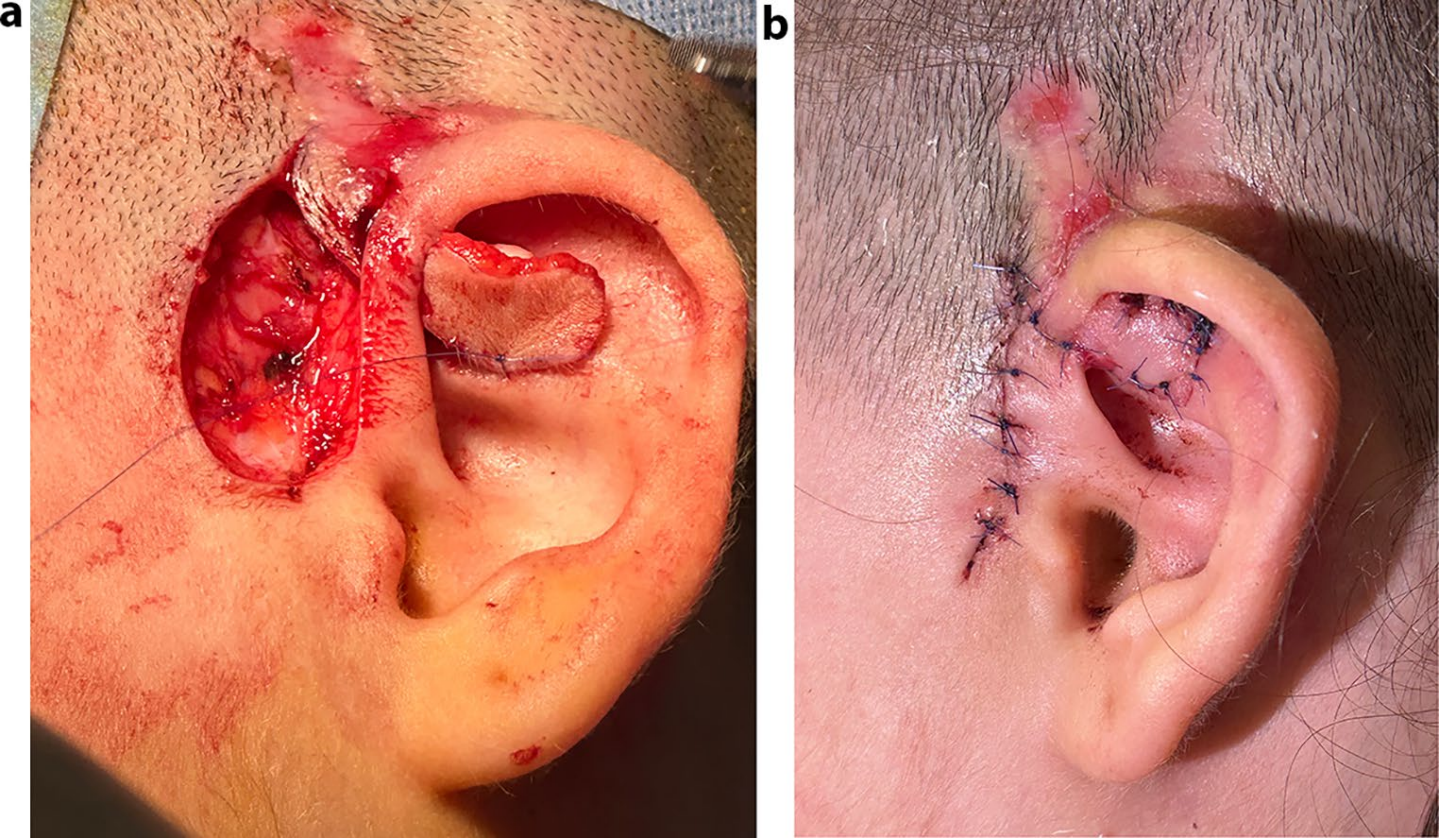
**a**: Closure of the defect with a single flap prepared in the preauricular region. **b**: Reinforcement with tragus cartilage

However, two months postoperatively, the electrode became visible again under the thinned skin of the triangular fossa where cartilage had been harvested in the previous surgery (Fig. 3). Under general anaesthesia, the 1 × 1 cm excised area was closed with a preauricular single-lobe flap rotated towards the defect area (Fig. 4a), supported by cartilage harvested from the tragus (Fig. 4b). The patient received antibiotics for 3 weeks. The family was informed for necessary of close follow-up. The patient was followed for six months without any problems, but further follow-up was not possible as the patient immigrated to another country. At audiological evaluation six months after the patient’s last surgery, bilateral pure-tone thresholds at 250, 500, 1000, 2000, 4000, and 6000 Hz were measured as 30-30-30-35-35-40 dB HL, respectively, with a speech recognition threshold of 30 dB HL. Telemetry measurements showed that electrode impedances were within normal limits bilaterally. NRT thresholds for the right ear were recorded as 180-142-192 for the 1st, 11th, and 22nd electrodes, respectively. In the left ear, no NRT responses were obtained for the 1st, 2nd, and 3rd electrodes during testing at all frequencies, while responses were recorded for the remaining electrodes. The NRT thresholds for the 6th, 10th, 16th, and 22nd electrodes on the left side were 184-191-160-139, respectively. When the right and left ears were assessed separately, the free-field pure-tone thresholds were as follows: Right ear: 35-30-30-35-35-40 dB HL at 250, 500, 1000, 2000, 4000, and 6000 Hz, respectively. Left ear: 35-35-30-35-35-40 dB HL at 250, 500, 1000, 2000, 4000, and 6000 Hz, respectively. The speech recognition threshold was 35 dB HL for both the right and left ear. Speech discrimination was 68% for both ears.

## Discussion

The revision rate for cochlear implant surgery has been reported to range from %2.6 to %8.3 in different studies [2–4]. Device malfunction is the most common reason, while revision for flap infection has been reported to range from %0.27 to %2.3 [2, 4–6]. Advances in surgical techniques, such as minimally invasive techniques and the avoidance of large incisions, have significantly reduced the need for revision due to flap infection [2, 5].

In cases of flap problems with skin loss, particularly with biofilm formation, the preferred approach is to remove the receiver portion of the device under the skin, leaving the intracochlear portion intact. After healing, reimplantation is performed. However, this approach requires the procurement of a new implant at additional cost.

Challenges demand innovative solutions. Our case was a refugee whose initial implants had been provided by the national health service, but who had no access to a new device for revision. Given the financial constraints, the problem was solved with a cartilage-reinforced flap that allowed continued hearing function in the affected ear. The six-month follow-up showed no further problems. The resistance provided by the cartilage prevented the electrode from extruding through the skin, solving the problem without the need for a new implant. Long-term follow-up was not possible as the patient moved to another country.

The implant used in this case, Nucleus Cl422, has a design where the ground electrode sits on top of the main electrode, increasing the thickness in this area. To solve such problems without recurrence, a deep channel is created during surgery to bury the bulkier part of the electrode in the bone. However, patient-specific anatomical challenges such as insufficient bone thickness or too anteriorly positioned sigmoid sinus can limit this approach. Implant manufacturers have addressed this issue by redesigning the electrodes to reduce the relative thickness at the point where the ground electrode exits the receiver. This modification prevents the recurrence of similar problems.

## Conclusion

In certain situations it may be necessary to develop solutions outside of established protocols. In this case, while the standard approach would be to remove the implant, create a new implant bed and perform a revision surgery with a new implant, the patient’s inability to obtain a new device necessitated the preservation of the existing implant. This approach successfully resolved the problem and prevented the patient from losing access to sound.

## Declarations

### Previous presentations

The case report was presented in part as a poster at the 45th Turkish National Otorhinolaryngology Head and Neck Surgery Congress, 23-27 October, 2024, Girne, Cyprus.
